# Contactless body temperature assessment for signalling humane endpoints in a mouse model of sepsis

**DOI:** 10.1017/awf.2025.10

**Published:** 2025-02-25

**Authors:** Catarina Miranda, Liliana Oliveira, Alexandre M Carmo, I Anna S Olsson, Nuno H Franco

**Affiliations:** 1 Bavarian Nordic; 2 i3S - Instituto de Investigação e Inovação em Saúde, Universidade do Porto; 3 IBMC - Instituto de Biologia Molecular e Celular, Universidade do Porto

**Keywords:** Animal welfare, humane endpoints, hypothermia, mice, sepsis, subcutaneous temperature, thermography

## Abstract

Minimising suffering is an ethical and legal requirement in animal research. This is particularly relevant for research on animal models of sepsis and septic shock, which show rapid progression towards severe stages and death. Specific and reliable criteria signalling non-recovery points can be used as humane endpoints, beyond which a study cannot be allowed to progress, thus preventing avoidable suffering. Body temperature is a key indicator for assessing animal health and welfare and has been suggested to have potential for monitoring the status of mouse models of sepsis. In this study, we monitored temperature variations using contactless methods – thermal imaging and subcutaneously implanted PIT tags – in a surgical model of sepsis by caecal ligation and puncture (CLP). We monitored body temperature variation following mid-grade CLP, high-grade CLP and sham surgery. All mice (*Mus musculus*) were monitored four times per day in the high-grade CLP model and three times per day in the mid-grade CLP model by both PIT tag readout and infrared thermography for ten days post-surgery, or until animals reached a predefined humane endpoint. Thermal data were compared with the clinical score and weight loss threshold used at our facility. Mean body surface temperature (MBST) assessed by thermal imaging and subcutaneous temperature (SCT) measured by PIT tags correlated, albeit not strongly. Moreover, while MBST does not appear to be a reliable predictor of non-recovery stages, SCT showed promise in this regard, even surpassing the widely used weight loss criterion, particularly for the high-grade CLP model of induced sepsis.

## Introduction

Humane endpoints play an important role in mitigating unnecessary suffering in laboratory animals, especially in progressive diseases and where other refinement measures are not effective (Franco *et al.* 2012). When successfully implemented, humane endpoints can enable terminating experiments before animals experience severe harm, without compromising scientific objectives. However, implementation of humane endpoints is often interpreted as the euthanasia of moribund animals. Such endpoints occur when the animal is euthanased upon becoming lethargic, unresponsive, and/or severely cachectic. And, while near-death endpoints prevent loss of biological samples due to autolysis between animal death and sample collection, from an animal welfare perspective they do not differ much from spontaneous death as an endpoint, as they fail to alleviate the progressively severe suffering preceding it (Franco *et al.* 2012). EU legislation requires avoiding death as an endpoint as far as possible (European Commission 2010). Biomarkers and clinical signs which reliably reflect disease severity (including predicting death) should thus be used as surrogate endpoints, whenever feasible (Franco *et al.* 2012).

Animal models are critical in advancing our understanding of sepsis, a complex and highly variable condition characterised by systemic inflammation and organ dysfunction. The caecal ligation and puncture (CLP) model replicates the clinical and pathophysiological features of polymicrobial sepsis by inducing peritonitis from a puncture in the caecum, leading to a cascade of inflammatory responses similar to those observed in human sepsis patients (Remick *et al.* 2000; Rittirsch *et al.* 2009). Moreover, the CLP model can reproduce different levels of sepsis, from mild to lethal, by varying the extent of caecal ligation and the number of punctures, making it a versatile tool in sepsis research (Mai *et al.* 2018).

In research on severe diseases such as sepsis, there is a conflict between the research purpose, requiring animals to manifest disease, and the duty to safeguard animal welfare. Disease-specific refinement measures, such as early humane endpoints, are important to mitigate this conflict (Nemzek *et al.* 2008; Lilley *et al.* 2015; Mai *et al.* 2018). However, when it comes to experimental sepsis, specifically the CLP model, clear endpoint markers have yet to be established (Mai *et al.* 2018). In the absence of an objective and accurate endpoint marker, researchers and animal care staff rely upon clinical score-sheets, posing a large risk that animals are removed from the study either too late, leading to unnecessary animal suffering, or too early, resulting in an incomplete number of observations and an increase in the number of animals subjected to this procedure (Drechsler *et al.* 2015), both of which raise ethical concerns. For this reason, surrogate markers of death have been proposed (Morton 2006; Franco *et al.* 2012), which typically rely upon clinical assessment, pain scales or other estimates of suffering to assess when euthanasia is appropriate (Shrum *et al.* 2014).

In a clinical setting, and similarly to what is observed in all deregulations of systemic inflammation, sepsis is accompanied by pronounced temperature abnormalities, manifesting as either fever or hypothermia. A similar scenario is expected to be found in experimental models of sepsis in mice, be it by CLP (Mai *et al.* 2018) or injection of an endotoxin (Remick *et al.* 2000; Franco *et al.* 2019). Among the physiological parameters used for measuring health and welfare in laboratory animals, body temperature is one of the most informative (Hunter *et al.* 2014; Mai *et al.* 2018; Mei *et al.* 2018). Hypothermia in particular has been shown to be an effective, clinically relevant method for monitoring disease progression and signalling non-recovery stages (Toth 2000; Trammell & Toth 2011; Mei *et al.* 2018), and thus potentially applicable for signalling humane endpoints in experimental sepsis (Laitano *et al.* 2018; Mai *et al.* 2018).

If body temperature is to be included as a humane endpoint criterion, it is important to measure it using technology that is easy-to-use, non-invasive, and provides accurate results. One such approach is infrared thermography (IRT), which can be applied non-invasively and even contactless in laboratory animals. To minimise detection bias in thermal image analysis, a software application – ThermoLabAnimal – for automatic mean body surface temperature (MBST) estimation of freely moving, group-housed mice (*Mus musculus*) has been tested and validated on mouse models of sepsis by high-dose LPS injection (Franco *et al.* 2019). Another monitoring technology option is RFID (radio frequency identification), which creates the opportunity for home-cage monitoring thereby minimsing operator bias and the impact of stress on the readout (Bartelik *et al.* 2024).

The primary objective of the current study was to identify temperature cut-off points that could be used to signal non-recovery states in surgically induced sepsis in mice by CLP, based on non-invasive temperature monitoring by both thermal imaging and the readout from thermosensitive RFID passive integrated transponder (PIT) tags, implanted subcutaneously. The goal was to ascertain whether these methods could be used to identify a hypothermia threshold that could signal non-recovery stages and be used as humane endpoints at an earlier stage than the current ‘moribund’ endpoint, based on a clinical score.

## Materials and methods

### Experimental design

Following the 3Rs principle, data for the present study were collected within another study testing the role of CD5L as a potential therapeutic for sepsis, namely comparing (A) the response to mid-grade experimental sepsis between wild-type (WT) and CD5L-knockout (CD5L-KO) mice (‘house mouse’), and (B) the response to severe experimental sepsis in WT mice treated with recombinant CD5L versus untreated controls. The results from the whole study, of which a subset of the sample was monitored for the purposes of this study, are presented elsewhere (Oliveira *et al.* 2024). We followed disease progression during each of the two aforementioned studies. Given it is a surgical model, few animals could be involved at a time. Thus, dividing each experiment into three batches of animals, with two animals per sex per genotype per batch for Study A on the mid-grade severity model. This meant n = 8 animals (two male WT mice, two male CD5L-KO mice, two female WT, two female CD5L-KO, all undergoing CLP) per batch, i.e. six males and six females per genotype, in an overall sample size of n = 24 mice.

For Study B, using the more severe model, three groups were planned: CLP mice treated with CD5L, CLP untreated mice, and sham-operated mice (caecum exteriorised and returned to the abdominal cavity neither ligated nor punctured). Therefore, three animals per condition per batch were used, thus n = 9 animals per batch and per treatment group (n = 27, overall). We followed disease progress for each batch by assessing body temperature (via three different methods, and later just two), weight variation, general locomotor activity, and other clinical signs that were registered into a clinical score-sheet for surgical models used at the animal facility.

### Study animals, housing and husbandry

The high-grade CLP model (or sham surgery) was carried out in females, 8 to 12 week old C57BL/6 WT mice, while the mid-grade severity CLP model was carried out on both male and female WT and CD5L-KO, 8 to 12 week old mice on a C57BL/6 background. CD5L-KO mice were generated by CRISPR/Cas9 engineering through the insertion of three stop codons in one of the first exons of the *Cd5l* gene) and control WT mice, as detailed elsewhere (Oliveira *et al.* 2024). Overall, 51 C57BL/6 mice were used during this study: 27 mice for the mid-grade severity sepsis model and 24 for the high-grade CLP sepsis model. We note that the classification of CLP as high- or mid-grade does not reflect the 2010/63/EU severity classification framework, but rather the magnitude of septic insult. Indeed, while the rhythm of disease progression differs between the two types of CLP, animals intervened by either can reach the severe classification, if they reach later stages of septic shock.

Animals were group-housed in single-sex groups in type II polycarbonate cages (268 × 215 × 141 mm [length × width × height]; floor area: 370 cm^2^), with absorbent autoclaved bedding, absorbent nesting material, and a cardboard tube. The temperature at the animal facility was maintained between 21–22ºC and humidity between 50–60%. Animals had *ad libitum* access to autoclaved food pellets and water. Mashed humid food was provided to animals unable to reach the food hopper and a fortifying supplement (Anima-Strath®, Switzerland) was supplied to animals presenting overt anaemia.

All procedures were conducted in accordance with the Decree-law 113/2013, which transposes the 2010/63/EU Directive, approved by institutional ethical review committees (i3S Animal Welfare and Ethics Body and the Competent Authority- DGAV) and conducted under the authority of the Project Licence (DGAV project licence 009951/2018-05-17). All animal work was performed at the i3S Animal Facility, which is licensed by the the Direcção Geral de Alimentação e Veterinária (DGAV) and accredited by AAALAC-International, meeting all current regulations and standards.

As a measure to promote transparency and higher reproducibility of results, this study was pre-registered at the Animal Study Registry (www.animalstudyregistry.org) (DOI: http://doi.org/10.17590/asr.0000206), time-stamped on January 27th, 2020. There were two small deviations from the pre-registered protocol. One is the absence of a reference to randomised batch designs, as the receiver operator curve analysis statistical analysis does not contemplate blocking for random variables. The planned blocks are therefore referred to here as batches. The second is that tail temperature measurement was abandoned, as it introduced more animal stress than anticipated, affecting both animal welfare and the temperature readout, as further explained below.

### Experimental procedure

**Figure 1.**
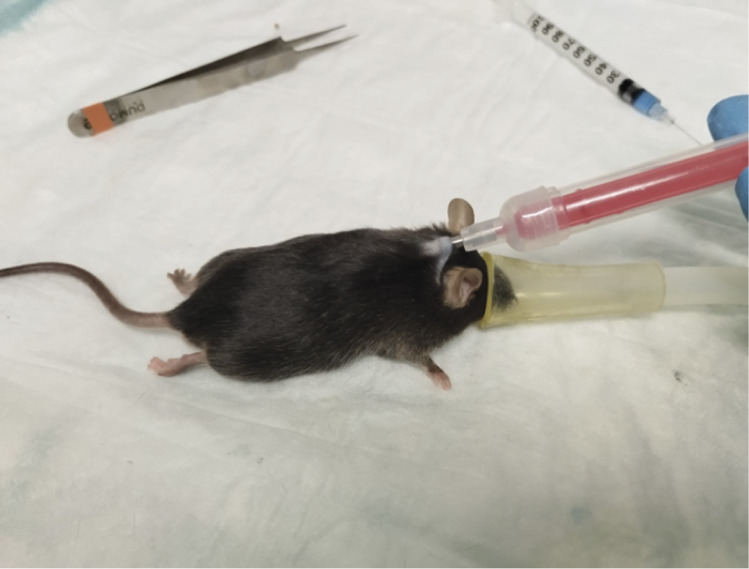
PIT tag implantation. Subcutaneous injection of a C57BL/6 mouse under Isoflurane anaesthesia, with a 13 × 2.1 mm (length × width) thermosensitive PIT tag, under the scruff region. On the top right corner, a syringe with surgical glue to shut the orifice made by the 12-gauge needle, and on the top left corner the tweezers used to join the adjacent tissues. Photograph courtesy of Nuno Henrique Franco.

All surgeries were performed by the same person (LO) on two consecutive days, with animals assigned in random order (by MS Excel® RAND function) to each surgery slot, in each batch. For pre-emptive analgesia, buprenorphine was injected subcutaneously (0.08 mg kg^–1^). Anaesthesia was induced in an induction chamber with 5% isoflurane and 1 L min^–1^ oxygen. Upon loss of righting reflex, mice were removed from the chamber and anaesthesia was maintained by a nose mask inhaling 3% isoflurane with 0.2 L min^–1^ oxygen. To determine anaesthetic depth, the loss of the pedal withdrawal reflex (i.e. no flexion of an extremity as a response to a pinch in a hind foot) was tested. During surgery, mice were placed on a heating pad and positioned on their backs. The lower quadrants of the abdomen were shaved and disinfected with a pad soaked in iodopovidone and alcohol at 70%, repeated three times. Ophthalmic ointment was applied to both eyes of the mice. During this procedure, glass-coated, 13 × 2.1 mm (length × width) thermosensitive PIT tags, (Biomark® BioTherm13 [USA], temperature reading range: 33ºC to 43ºC; 0.1ºC sensitivity; factory calibrated), were preloaded into a 12-gauge needle, and implanted subcutaneously in anaesthetised animals prior to CLP, into the loose skin over the interscapular region. The puncture site was closed with a small drop of surgical glue (Vetbond®, USA) (Figure 1).

The CLP procedure was performed according to guidelines by Rittirsch *et al*. (2009), as illustrated in Figure S1 (see Supplementary material). To minimise trauma, a small longitudinal skin midline incision measuring approximately 1.5–2 cm was made using dissection scissors. A vertical midline incision was made through the translucent *linea alba.* After the intermuscular, fascial, and peritoneal layers were sectioned using blunt forceps, the caecum was located and exteriorised. With the assistance of a swab, the caecal contents were pushed gently toward the distal caecum to ensure there was no air or gases trapped inside the caecum before ligation. The severity grade is dependent on the position of caecal ligation. In the high-grade severity model, the distance between the distal pole and the ligation basis of the caecum was approximately 70–75% of the caecum, while for the mid-grade model the portion of the ligated caecum was roughly 40–50%. The caecum was then perforated with a single through-and-through puncture equidistant to the tip of the caecum and the ligation site, with a 21-gauge needle, taking caution not to puncture any blood vessels. Finally, the bowel was returned to the abdominal cavity and the peritoneum, fasciae, and abdominal musculature closed with a simple continuous polyglycolic acid suture. The exterior skin layer was closed with metallic clips and pre-warmed saline solution (0.9% NaCl) was injected (5 ml 100 g^–1^ of bodyweight) subcutaneously.

For postoperative care, buprenorphine analgesia was repeated every 12 h for two days after the surgery. After surgery, mice were placed inside a cage over a heating pad and provided with sheets of absorbent paper until they had fully recovered from the anaesthesia. Afterwards, animals were returned to their original cage, and for the entire duration of the study, the cages were partly placed over a heating pad (half on top of it, the other half out), following the standard operating procedure at the animal facility. The heating pad was not removed in order to ensure the temperature within the cages remained constant for the entirety of the study and was similar for all animals.

### Monitoring scheme

**Figure 2.**
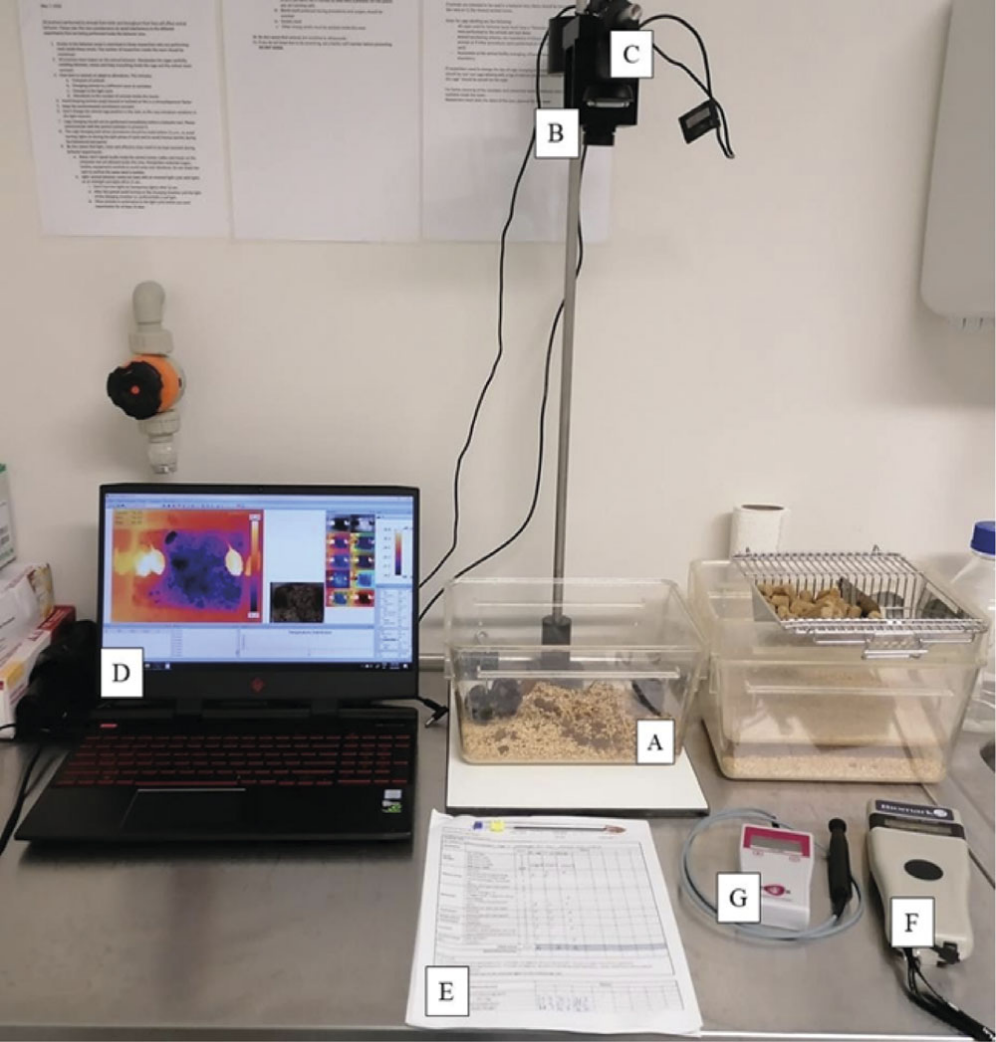
Experimental set-up. Photograph depicts the materials used for this study, which include a cage with the animals being monitored (A), a thermal camera mounted above the cage (B), a visible light camera for taking pictures of the animals concomitantly with the thermal camera (C), the thermal camera software, depicting images from both cameras, where the thermogram of the group of animals in the cage is highlighted (D), the score-sheets where the study animals’ clinical scores were registered several times a day (E), the hand-held PIT-tag reader (F), and an infrared tail thermometer which, despite being designed for use in mice, was deemed detrimental to animal welfare and the quality of results, due to the evident stress-induced hyperthermia it elicited (G). Photograph courtesy of Catarina Miranda.

After surgically induced sepsis, body temperature was monitored at fixed time-points. The animals induced with the high-grade CLP model were monitored four times a day, whereas the mice induced with mid-grade CLP model were monitored three times a day. In both cases, they were monitored for ten consecutive days. Monitoring frequencies were higher for the first batch of animals to gain an understanding of variation dynamics and afterwards defined for the aforementioned schedule as a result of observed temperature change patterns. Three different methods were originally used to assess the course of the disease progression: Thermal image acquisition of animals in the cage by IRT; Assessment of subcutaneous dorsal temperature from readout of thermosensitive PIT-tags using a handheld Biomark GPR+ reader; and Tail temperature as assessed by a single-point infrared thermometer (all equipment and set-up represented in Figure 2). Tail temperature rose substantially within seconds of containing the animal, due to a hyperthermic stress response (Blenkuš *et al.* 2022). This method was hence found to lack accuracy and reliability with its use subsequently discontinued. Clinical signs (e.g. posture, activity) were assessed at each monitoring session, using the clinical score-sheet for post-surgical monitoring in use at our institution. The following attributes were scored on a severity scale from 0 (normal) to higher values: body weight, appearance, behaviour, hydration, respiration, incision status, and automutilation. Intervention thresholds were set as: 1–4 (increased monitoring, consider analgesia), 5–9 (intensive monitoring, analgesia, veterinary input), and > 10 (consider humane endpoint). It was not possible for either observers or animal care staff to be blinded since the animal facility staff required each procedure animals underwent to be clearly identified in each cage.

Humane endpoints were applied to avoid death as an endpoint. Criteria were based on a predefined score or reaching bodyweight loss higher than 20%, regardless of the clinical score. Animals reaching the humane endpoint were euthanased by CO_2_ asphyxiation.

### Thermal images acquisition

An infrared thermal camera (‘Thermal Expert’ TE-EV1 Camera, South Korea) and a visible-light digital camera (Microsoft LifeCam HD-3000, China) were fixed on a specially devised structure, for a birds-eye perspective. Both cameras were set-up 60 cm from the table. The camera was switched on in the morning, approximately 40 min prior to the first measurement and left running for the entire day. Thermal Expert Q1 1.8.1. software was used to capture the thermal images.

**Figure 3.**
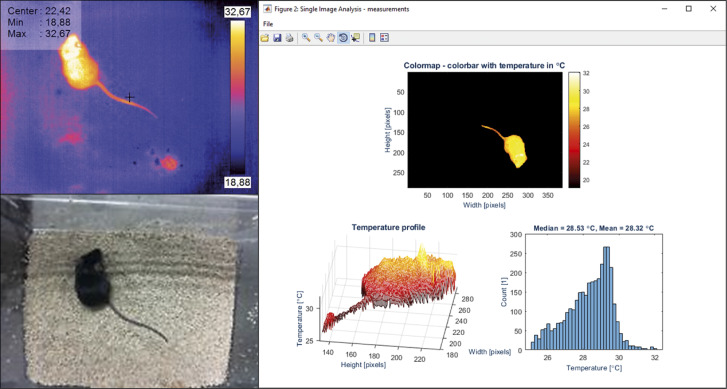
Thermal image acquisition and analysis. The bottom left corner shows the visible-light digital camera footage with the the top left corner showing the thermal image acquired by the thermal camera, both taken using the Thermal Camera native software. The large image on the right shows the automated output provided for each thermal image by the in-house developed ThermoLabAnimal software. This removes the thermal background (top, note the vertical and horizontal axis image flipping), provides a 3D thermal graph (left) with temperature as the ‘third dimension’ as well as a thermal histogram of the thermogram of the cropped animals, with mean and median temperatures (in this case, of the group of animals in the frame).

Each mouse was removed from the home cage, transferred into a clean cage, and placed under the cameras for thermal image collection. Three images were captured for each measurement. Each measurement took a few seconds. The thermal images obtained were analysed by ThermoLabAnimal (Franco *et al.* 2019), a dedicated software package for automatic, high-throughput mean body surface temperature (MBST) estimation (previously established as a robust parameter; Gjendal *et al.* 2018). The software analysed all images automatically (removing the possibility of observer bias), taking away the background and providing the mean temperature for each animal (Figure 3).

The Resource Equation (Mead 1988, cited in Festing 2014), for sample size estimation was considered an acceptable approach for calculating sample size for the immunology of sepsis study (from which we collected data for this one). Briefly, for an unblocked design, E = (total number of experimental units) – (number of treatments), E should be between about 10 and 20, “with some leeway” (Festing 2014). For a sample size of n = 24 (E = 24 experimental units – 4 treatment groups = 20) for the high-grade CLP, and n = 27 for the study on the mid-grade CLP (E = 27 – 3 = 24), a power calculation for s signed ranks test estimates that differences between a 75% sensitivity (or specificity) from 50% or below can be identified as significant, with 80% power (β = 0.2) and α = 0.05. A sensitivity/specificity of 50% (or below) corresponds to the null hypothesis since it means cut-off points would not have better predictive value than the toss of a coin. In any case, the expected high prevalence of the condition (in this case, animals reaching the humane endpoint) means that relatively small samples could be considered sufficient.

The predictive value of each of these parameters was compared, based on their ability to signal non-recovery, for each of the CLP models. The ideal temperature cut-off point was then determined by ROC (receiver operator curve) analysis, based on the higher value for Youden’s J index.

## Results

**Figure 4.**
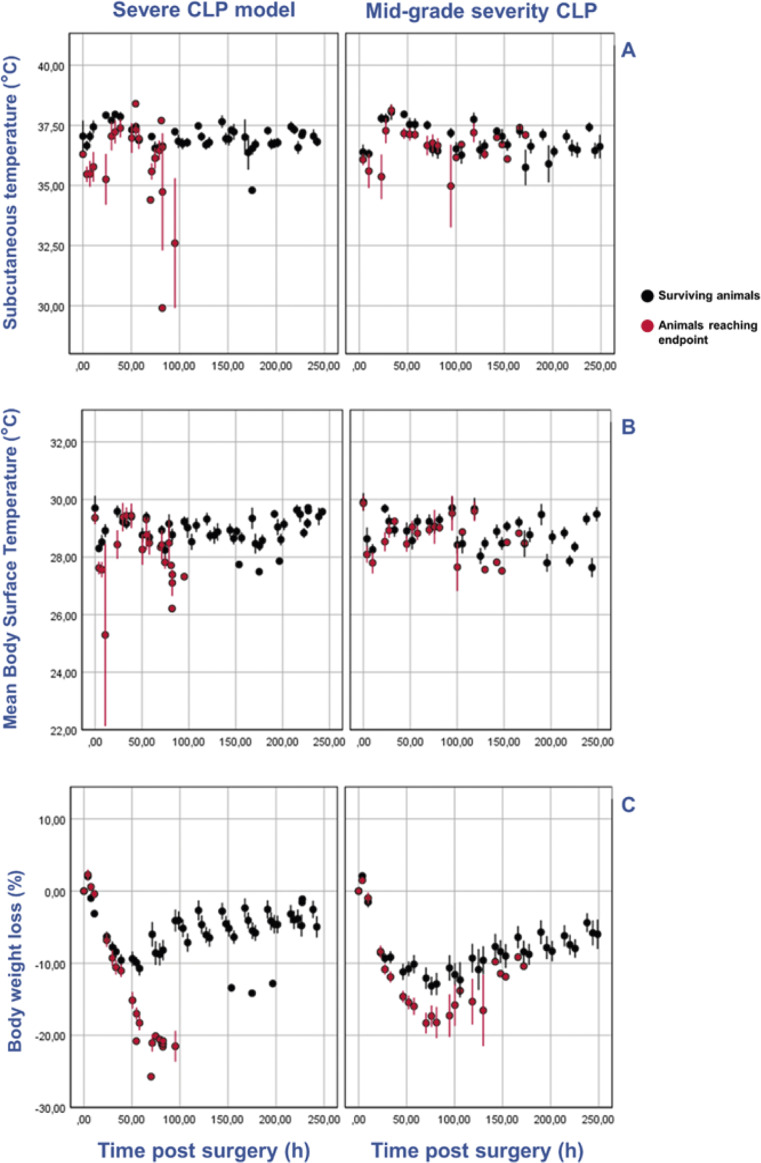
Body temperature and weight variation following surgery. This six-panel image shows clinical data retrieved after high-grade caecal ligation and puncture (CLP) (left column) or mid-grade severity CLP (right column), namely subcutaneous temperature (SCT) from PIT-tags readout (A - top row), mean body surface temperature (MBST) assessed by a thermal camera (B - middle row), and mean percentage of weight loss (C - bottom row). Of the wild type mice enrolled in Study B (n = 27, left column), n = 18 underwent high-grade CLP and n = 9 sham surgery, and mean body surface temperature (MBST) (obtained by thermal imaging) and subcutaneous temperature (SCT) were obtained four times per day for ten days. For Study A (right column), CD5L-KO mice (n = 12) and WT mice (n = 12) underwent mid-grade CLP, and MBST and SCT were obtained three times per day for ten days. Dots and bars in red represent mean (± SD) for animals reaching the humane endpoint according to a clinical score-sheet used at our facility, and in those animals surviving for the duration of the study.

A decrease in both mean body surface temperature (MBST) and mean subcutaneous temperature (SCT, as measured by PIT tag readout) was observable in animals with CLP-induced sepsis to which a humane endpoint was applied, compared to mice that survived up to the ten-day observation period, in particular in the last measurements before endpoint (Figure 4). The mean MBST of high-grade CLP-induced animals was the lowest 29 h post-surgery (25.20°C), with SCT reaching the lowest 73 h post-surgery (falling below the reading range of the PIT tag reader of 33.0°C and registered as 32.9°C). Overall, the temperature of the animals reaching the humane endpoint appears to be lower than that of the animals that survived, although the difference appeared more prominent for subcutaneous temperature. A similar trend appears to be observable for the mid-grade CLP model. The mean temperature of the animals reaching the humane endpoint was lower than for the surviving animals, for both SCT and MBST, which recovered after a decrease in mean temperature, unlike what was observed for the high-grade CLP models.

A progressive and substantial mean weight loss following surgery was observable for high-grade CLP-induced animals that ultimately reached the humane endpoint (ten out of 26) and, as expected, the lowest value corresponded with the predetermined threshold (21% loss). Surviving animals also lost weight, although recovery was observable. The lowest values coincided for both survivors and non-survivors 73 h post-surgery. As would be expected, the lowest mean bodyweight loss for survivors (–11%) was still considerably higher than that of animals reaching the humane endpoint. For the mid-grade severity model, there was also a steep mean loss of bodyweight for animals reaching the endpoint (14 out of 24), with bodyweight being consistently lower than for survivors from 24 h after surgery, although a few non-survivors recovered some of the weight loss before reaching the humane endpoint.

As expected, MBST were found to correlate and predict each other significantly, albeit not strongly (R = 0.48; R^2^ = 0.23; *P* < 0.001).

**Figure 5.**
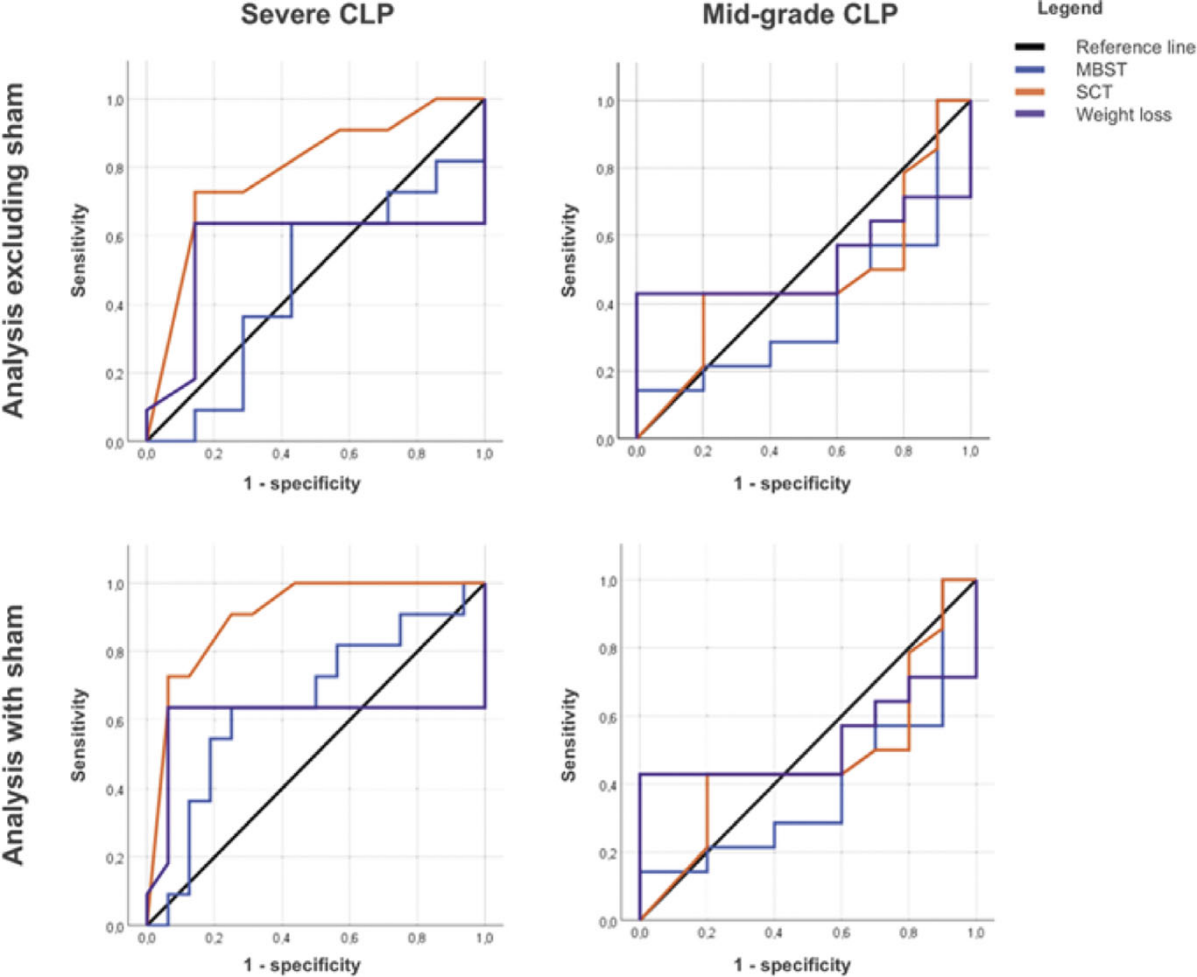
Receiver Operating Characteristic (ROC) curve analyses. This four-image panel displays the ROC curves for three clinical parameters, namely the lowest MBST (mean body surface temperature, in red), lowest SCT (subcutaneous temperature, green) and lowest body weight (yellow) as regards their sensitivity and specificity as predictors of death in two models of CLP (caecal ligation and puncture) induced septic mice. The curves for the high-grade CLP are displayed to the left, while for mid-grade severity CLP are represented on the right. The black line represents the reference line for statistical comparison in ROC analyses. The top graphs represent the analysis excluding sham-surgery animals, and the bottom one includes them. The ideal cut-off point was determined by ROC analysis, based on the higher value for Youden’s J index, shown on Table 1.

To investigate whether MBST could be used as a surrogate marker of death in the CLP model of sepsis, we carried out a Receiver Operating Characteristic (ROC) curve analysis for both high- (n = 27) and mid-grade severity CLP animals (total n = 24). We performed two analyses, one excluding sham-operated mice (as death is not an expected outcome) and a second analysis including sham-operated mice (as they underwent surgery and dropped body temperature on account of the general anaesthesia, and hence could provide further insight, as a basis for comparison). Output from both approaches is reported (Figure 5). The criteria used for ROC curve estimation were the lowest recorded MBST, the lowest recorded SCT and the lowest decrease in the percentage of lost bodyweight of initial weight for each animal.

In the ROC curve obtained without the sham animals with the high-grade CLP for weight loss, the highest Younden’s index was of J = 0.493, for –19.35% (63.6% sensitivity, 85.7% specificity). For MBST, the highest Younden’s index was of J = 0.207 (63.6% sensitivity, 57.1% specificity, for MBST lower than 26.67ºC). For SCT, setting the threshold for 34.4ºC (Younden’s index J = 0.584) for deciding upon when to euthanase animals would have a sensitivity of 72.7% with a specificity of 85.7%. Under the same conditions, for the mid-grade model for weight loss, the highest Younden’s index was of J = 0.43, for –20.54% (42.9% sensitivity, 100% specificity). For MBST, the highest Younden’s index was of J = 0.143 (14.3% sensitivity, 100% specificity, for MBST lower than 25.34ºC). For SCT, setting the threshold for 34.45ºC (Younden’s index J = 0.229) for deciding upon when to euthanase animals would have a sensitivity of 42.9% with a specificity of 80%.

The ROC curve obtained with the sham animals included for the high-grade model appeared to have the best overall model quality (Table 1). For weight loss, the highest Younden’s index was of J = 0.573, for –19.35% (63.6% sensitivity, 93.7% specificity). For MBST, the highest Younden’s index was of J = 0.386 (26.67% sensitivity, 75% specificity, for MBST lower than 25.34ºC). For SCT, setting the threshold for 35.25ºC (Younden’s index J = 0.229) for deciding upon when to euthanase animals would have a sensitivity of 90.9% with a specificity of 75%. The lowest predictivity was found for the mid-grade model. For weight loss, the highest Younden’s index was of J = 0.429, for –20.54% (42.9% sensitivity, 100% specificity). For MBST, the highest Younden’s index was of J = 0.143 (14.3% sensitivity, 100% specificity, for a MBST lower than 25.34ºC). For SCT, setting the threshold for 34.45ºC (Younden’s index J = 0.229) for deciding upon when to euthanase animals would have a sensitivity of 42.9% with a specificity of 80%.

**Table 1.** The area under the curve calculated for each of the putative predictors tested. A predictor with an area under 0.5 is typically deemed uninformative

Variables	Area under the curve			
	Analysis including sham		Analysis without sham	
	High-grade CLP model	Mid-grade CLP model	High-grade CLP model	Mid-grade CLP model
Subcutaneous	0.792	0.471	0.906	0.471
MBST	0.468	0.389	0.653	0.389
Weight loss	0.565	0.521	0.605	0.521

CLP: Caecal ligation and puncture;

MBST: Mean body surface temperature.

## Discussion

The purpose of this study was to investigate whether a low-temperature cut-off point would predict non-recovery stages, which in turn could be used as a proxy of spontaneous death or the moribund stage typically used as endpoint, in a murine surgical model of sepsis.

In animals subjected to CLP, there was an observable decrease in MBST and SCT after surgery, in both models, and for both outcomes. This is consistent with the literature reporting hypothermia observed 4 to 32 h after induction of CLP (Granger *et al.* 2013; Li *et al.* 2018; Mai *et al.* 2018).

Overall, across all measurements, on both models, the temperatures of surviving animals were higher than the temperatures of the animals reaching the pre-established humane endpoint, as previously demonstrated (Mai *et al.* 2018), although this report found more pronounced body temperature drops for animals reaching the endpoint than the ones we observed. A likely explanation might be found on the heating pad kept under half of the cage for the entire duration of the study, to maintain a constant environmental temperature within the cages, as imposed by the animal facility once it was understood that animals could be chronically hypothermic. The cage temperature is a contributing factor in differences in temperature between an animal surface temperature and core temperature. Subcutaneous temperature can vary widely, and is strongly affected by the ambient temperature (Meyer *et al.* 2017), with surface temperature measurements having higher degrees of variation, with a significant difference in temperature between core and surface temperature resulting from cage temperature (Mei *et al.* 2018). This raises the question of the consequences to the research output of heating the cage floor as a refinement measure, which will depend upon whether hypothermia should be treated as a secondary consequence of sepsis – and therefore mitigated as one would expect in a clinical setting – or as a primary outcome informing on pathology. In the latter case, the possibility of skewing results should be weighed against the welfare benefits of addressing severe hypothermia. It should be noted, however, that even having part of the cage over a heated pad to where animals could seek warmth, hypothermia (even severe hypothermia) was still overtly observable in animals with more severe clinical signs.

Mice subjected to the high-grade CLP procedure presented lower body temperature across all the measurements when compared to mice undergoing the mid-grade CLP model, similar to previous studies (Mai *et al.* 2018).

Animals subjected to CLP presented lower mean temperatures and more abrupt variations than sham-operated animals. In both cases, there was a slight decrease in the first measurements after the surgery, but the sham-treated mice quickly recovered, also as previously reported (Ebong *et al.* 1999; Mai, *et al.* 2018). The temperature in animals submitted to sham surgery remained relatively stable, aside from the expected circadian variations, also previously reported (Gordon 2012) both for telemetry and thermography data across the circadian cycle, with periods of relative stability over limited times of this cycle. One of the limitations of measuring sham-operated animals is that some of the animals removed their PIT tags. In fact, 44% of these animals had removed their tags by the end of the experiment, a phenomenon not observed in CLP mice. This difference is likely due to the CLP procedure’s impact on mice, resulting in these mice being less active and less responsive. Measuring body surface temperature using infrared thermography posed no such limitations.

We found body surface temperature and subcutaneous temperature to be correlated, albeit not strongly. Those findings are contrary to previous studies (Nemzek *et al.* 2008; Mei *et al.* 2018) that found a correlation between core and surface temperatures using infrared thermometers, but explainable by reports stating that correlation between surface temperature and core temperature can be consistent within each mouse, but very idiosyncratic to each individual (van der Vinne *et al.* 2020).

The 90.9% sensitivity and 75% specificity for subcutaneous temperature below 35.25ºC suggests this parameter holds potential as a complement to the percentage of weight loss. This is further highlighted by previous findings suggesting that temperature could be an early indicator of impending death (Nemzek *et al.* 2008; Mai *et al.* 2018), as a clinically relevant method for monitoring and predicting non-recovery stages. On the other hand, the idiosyncratic nature of the relationship between core and surface temperature for each individual mouse (van der Vinne *et al.* 2020) might make thermography an unreliable method as a humane endpoint, given that measurements are compared to a reference value, rather than to individual variations. Also, standard PIT tag readers do not read temperatures below 33ºC (as temperatures read outside the 33–43ºC range may be skewed), and these were observed in some animals (signalled by the reader as ‘TEMP BELOW RANGE’). This fact possibly affected the correlation analysis, given that 32.9ºC had to be used as a surrogate value for SCT. However, the use of thermosensitive PIT tags has important advantages worth exploring, namely the possibility of automated, continuous assessment through home-cage monitoring, minimising operator bias and the impact of stress on the readout (Bartelik *et al.* 2024).

Other factors may have affected our data, given that clinical assessment and all the measurements were made in a different room from where the animals were housed. Carrying the cages from room to room might have caused increased stress, resulting in stress-induced hyperthermia within minutes (Zethof *et al.* 1994; Blenkuš *et al.* 2022), for those animals still capable of generating heat. More often than not, however, we observed a drop in body temperature, suggesting that chronic hypothermia had more impact than any potential short-term, stress-induced hyperthermia, which moreover requires physiological fitness. As for the transient (lasting seconds) peripheral vasoconstriction in response to acute stress (Blenkuš *et al.* 2022), as often observed in the tail, this was unlikely to have had a relevant impact on the measurements, as the tail bears little influence on the overall MBST, due to its relative size. Also, in hypothermic animals, it often failed to even show up in the thermogram, as peripheral vasoconstriction is a typical physiological response to hypothermia, to reduce heat loss.

Based on historical data, a research group recently determined weight loss as a good predictor of the clinical score-based humane endpoint for intranasal infection models with *Streptococcus pneumoniae* and H1N1, as well as systemic infection models with *Candida albicans* and *Listeria monocytogenes*, proposing however that thresholds should be adapted to each model in question, and therefore not be applicable universally (Brochut *et al.* 2024). In our study on CLP-induced sepsis – an acute and rapidly progressive, multi-pathogen infection, weight loss was not found to be predictive of outcome. Weight loss was indeed less predictive of the humane endpoint than expected, despite being in itself a parameter for clinical scoring and a loss higher than 20% being sufficient for applying a humane endpoint at our facility, regardless of other clinical signs. While this would, in theory, strongly bias prediction towards this parameter, no such artefact was found, or at least it did not have a sufficient impact to make it a good predictor. This was likely due to several CLP-induced septic animals reaching a clinical score warranting euthanasia despite not reaching severe weight loss.

### Animal welfare implications

This study highlights the potential for subcutaneous temperature measured by thermosensitive PIT tags as a reliable, minimally invasive biomarker for signalling humane endpoints in a mouse model of sepsis. The use of SCT allows for early detection of non-recovery stages, reducing unnecessary suffering by facilitating timely euthanasia decisions before reaching severe moribund conditions. Compared to other conventional parameters, such as weight loss, SCT demonstrated higher sensitivity and specificity, particularly in the most high-grade CLP model of sepsis. Incorporating this hypothermia-based criterion in decisions about applying humane endpoints could help improve animal welfare in research protocol, while maintaining scientific rigor.

## Conclusion

Mean body surface temperature (MBST) proves less reliable for assessing temperature loss when cages are kept on a heating pad for the entire duration of the experiment. Weight loss, contrary to expectations, was also a less effective predictor of humane endpoints. In contrast, subcutaneous temperature measured by PIT tags demonstrates significant promise for reliably predicting non-recovery stages in a high-grade CLP-induced sepsis model, thanks to its high sensitivity and specificity. This highlights the advantages of using novel temperature measurement methods over bodyweight for determining humane endpoints.

## Supporting information

Miranda et al. supplementary material Miranda et al. supplementary material

## Supplementary material

The supplementary material for this article can be found at http://doi.org/10.1017/awf.2025.10.
