# Supplementary material for: Contactless body temperature assessment for signalling humane endpoints in a mouse model of sepsis
Source: Anim Welf. 2025 Feb 25;34:e13. doi: 10.1017/awf.2025.10 (PMC11867820; doi:10.1017/awf.2025.10)
Supplement: Miranda et al. supplementary material [file S0962728625000107sup001.pdf]

Supplementary materials

Contactless body temperature assessment for signalling humane endpoints in a mouse model of sepsis

Catarina Miranda[1], Liliana Oliveira[2,3], Alexandre M Carmo[2,3], I Anna S Olsson[2], Nuno H Franco https://orcid.org/0000-0003-4015-0532[2]

[1] Bavarian Nordic

[2] i3S - Instituto de Investigação e Inovação em Saúde, Universidade do Porto

[3] IBMC - Instituto de Biologia Molecular e Celular, Universidade do Porto

Author for correspondence: Nuno H Franco, email: nfranco@i3s.up.pt

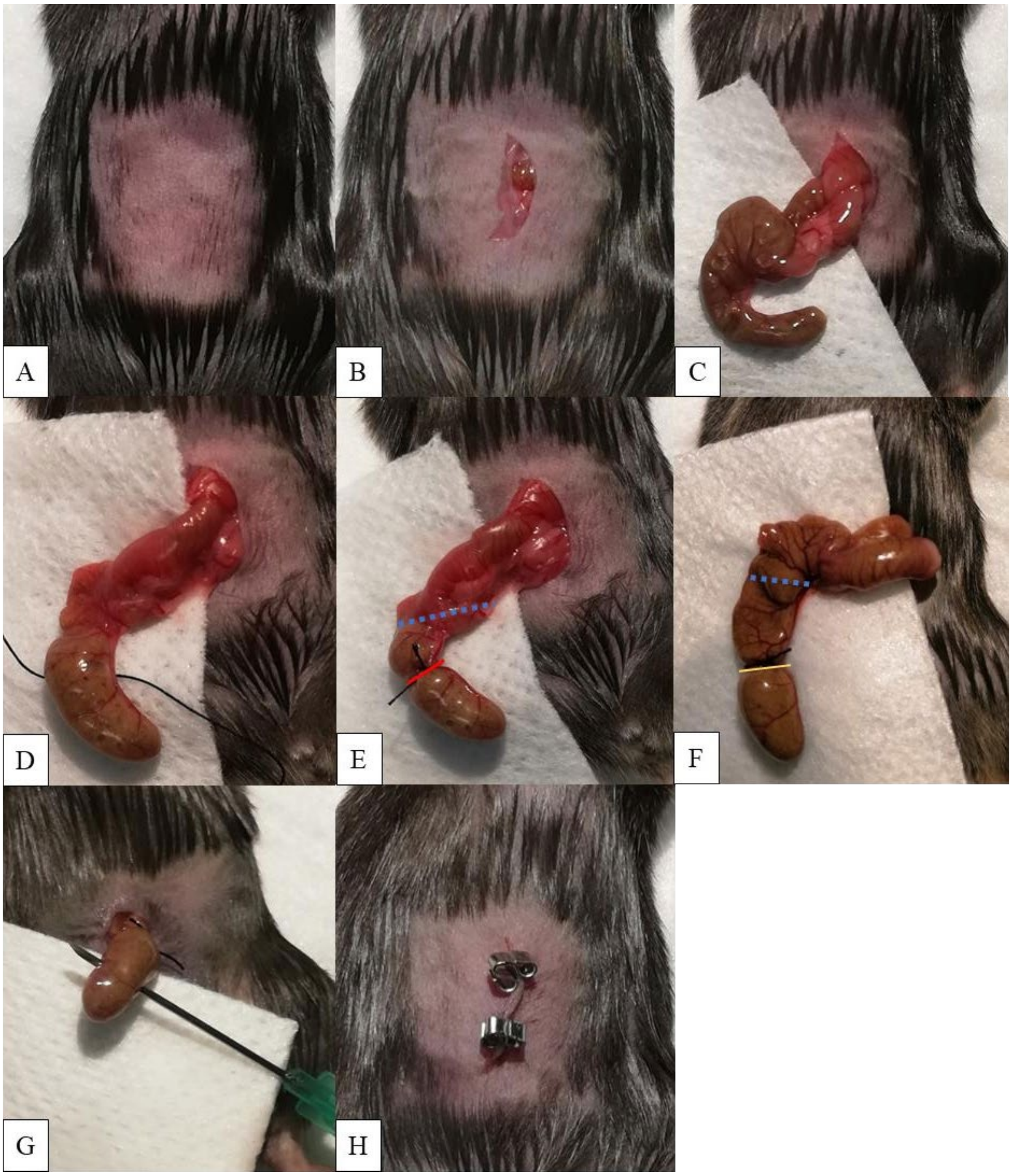

Figure S1. Experimental induction of sepsis by caecal ligation and puncture. This series of images depict the steps performed in this technique to induce polymicrobial sepsis, by ligating the caecum at a defined position and puncturing it with a needle. The extrusion of caecal contents leads to peritonitis and systemic infection, mimicking clinical manifestations of the disease. Photographs show (A) disinfected surgical area, (B) longitudinal skin and *linea alba* midline incision, (C) exteriorised caecum, (D) caecal contents pushed toward the distal caecum, (E) in the high-grade severity model, 70 to 75% percent of the caecum was ligated (indicated by the orange line) from the ileo-caecal valve (indicated by the dotted blue line), (F) in the mid-grade severity model, 40 to 50% of the caecum was ligated (indicated by the yellow line), (G) caecum perforated with a needle and (H) caecum returned to the abdominal cavity and incision site closed. Photographs courtesy of Catarina Miranda.
